# Impact of the health insurance deregulation policy for cross-regional healthcare on hospitalization visits and expenses of patients with ischemic heart disease: an interrupted time series analysis

**DOI:** 10.3389/fpubh.2025.1609842

**Published:** 2025-07-02

**Authors:** Yueying Cui, Xi Wang, Jiu Cheng, Yifei Wang, Huimin Yang, Ruihua Feng

**Affiliations:** ^1^Institute of Medical Information, Chinese Academy of Medical Sciences and Peking Union Medical College, Beijing, China; ^2^School of Government, Beijing Normal University, Beijing, China

**Keywords:** insurance deregulation, direct settlement, interrupted time-series analyses, ischemic heart disease, hospitalization expenses, accessibility

## Abstract

**Background:**

The health insurance deregulation policy aimed to enhance healthcare accessibility by eliminating intra-provincial administrative hurdles. However, its impact on hospitalization patterns of high-burden chronic conditions like ischemic heart disease (IHD) remains unexamined.

**Methods:**

Interrupted time-series analysis (ITSA) was employed to evaluate weekly hospitalization visits and expenses for 8,522 IHD inpatients across three Hebei counties (January 2021–July 2023). Models assessed immediate and longitudinal changes post-policy, adjusting for autocorrelation and seasonal trends.

**Results:**

Policy implementation triggered an immediate 20.27 surge in weekly hospitalizations (*p* = 0.006), with sustained utilization unaffected (β_3_ = 0.17, *p* = 0.619). Per-visit hospitalization expenses maintained pre-deregulation policy declining trends (−126.71 CNY/week pre-policy vs. −32.04 CNY/week post-policy), despite a non-significant instantaneously increase by 478.43 (*p* = 0.723) in the first week following the implementation of this policy. Additionally, the health insurance deregulation policy reversed the weekly trend of insurance reimbursement costs per visit from decreasing (−81.98 CNY/week) to increasing trajectories (1.95 CNY/week, *p* = 0.004).

**Conclusion:**

The health insurance deregulation policy successfully expanded IHD care access without exacerbating financial burdens, demonstrating that administrative simplification can coexist with cost containment under concurrent payment reforms.

## 1 Introduction

The equitable allocation of medical resources remains a persistent challenge in healthcare systems globally, with pronounced disparities often reflecting regional economic gradients. In China, this imbalance is evident in the concentration of advanced medical facilities and specialist expertise in economically developed provinces (1). Even within individual provinces, disparities in intraregional medical resource lead to patient migration from underserved areas to urban medical hubs. Government statistics reveal that cross-regional healthcare-seeking behavior is widespread in the country. In 2024, the nationwide number of cross-provincial direct settlement cases for inpatient and outpatient reached 14.34 million and 224 million respectively, accounting for 4.91% and 3.34% of the total inpatient and outpatient claims (2). However, the medical insurance coverage policies are different between the coordinated regions (insured jurisdiction) and out-of-coordinated regions. The coordinated regions mean the level of health insurance fund poll. Up to 2024, most of the health insurance fund pooling is at the municipal level in China. The tiered medical insurance system historically required patients seeking cross-regional care outside their coordinated regions to pay upfront costs and navigate complex post-treatment reimbursement procedures. This financial and administrative burden disproportionately impacts vulnerable populations, including those with low incomes, potentially delaying care and exacerbating health inequities (3).

**Figure 1:**
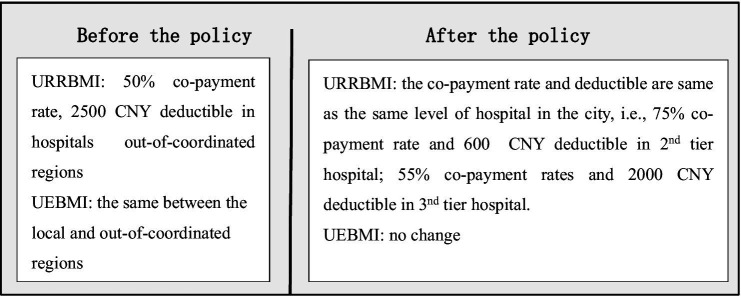
Health insurance reimbursement standards before and after the insurance deregulation policy for cross-regional healthcare for inpatients. URRBMI, Urban–Rural Resident Basic Medical Insurance; UEBMI, Urban Employee Basic Medical Insurance.

In response, China introduced direct settlement mechanisms for cross-regional healthcare to mitigate these barriers. While this reform eliminated upfront payments, it retained a digital record filing requirement through platforms like WeChat Mini Programs, Official Accounts, or mobile applications, to verify insurance eligibility and determine reimbursement tiers. Crucially, reimbursement rates for cross-regional care remained substantially lower than local treatment, creating persistent financial disincentives for care-seeking. On September 1, 2021, Hebei Province implemented a landmark policy reform by abolishing record filing requirements, which was referred to as an insurance deregulation policy, enabling patients to access same-tier hospitals within coordinated regions without reimbursement penalties. The health insurance reimbursement standards before and after this policy are detailed in Figure 1.

Existing literature presents conflicting perspectives on patient mobility effects. While some studies (4–6) warn of insurance fund risks from unrestricted cross-border healthcare, empirical evidence from China’s Yangtze River Delta^1^ reforms suggests outpatient utilization remained stable post-deregulation (7). One study showed that facilitating the greater patient choice does not necessarily stimulate potential demands (8). Most analyses (9, 10) focus on general populations or specific surgical procedures, leaving critical gaps in understanding chronic disease management. This oversight is particularly concerning for Ischemic Heart Disease (IHD), China’s second leading cause of disability-adjusted life years (DALYs), with national DALYs escalating from 159.9 million (2010) to 188.3 million (2021) (11). Concurrently, the hospitalization costs for IHD surged to 116.96 billion CNY (2020), with an average cost of 14,638 CNY per admission (12). The financial burden is further exacerbated by frequent readmissions and the need for long-term care (13).

Despite this growing crisis, no studies have systematically evaluated how healthcare policy reforms influence IHD care trajectories. Our interrupted time-series analysis aimed to addresses this gap by investigating both immediate and longitudinal effects of Hebei’s health insurance deregulation policy on hospitalization visits and expenses for IHD inpatients. Focusing on a high-burden chronic condition with complex care pathways, this study advances beyond previous mobility research limited to surgical volumes or aggregate utilization. Findings will inform evidence-based policy optimization for chronic disease management in fragmented healthcare systems.

## 2 Methods

### 2.1 Study design

This quasi-experimental study focused on Hebei Province, a region adjacent to Beijing and Tianjin, where high-quality medical resources are concentrated in northern China. The intervention analyzed was the health insurance deregulation policy implemented on September 1, 2021, which eliminated administrative barriers for cross-regional healthcare access within the province. Prior to this policy, patients seeking care outside their coordinated regions faced higher deductibles and reduced reimbursement rates under the Urban–Rural Resident Basic Medical Insurance (URRBMI). Post-policy, URRBMI enrollees could freely access same-tier hospitals across Hebei without reimbursement penalties, aligning intra-provincial reimbursement standards with local care.

The study focused on URRBMI-insured patients, who constitute 68.3% of China’s insured population in 2023 (14) and face higher out-of-pocket (OOP) expenses and catastrophic health expenditure risks compared to Urban Employee Basic Medical Insurance (UEBMI) enrollees. Individual-level inpatient claims data were extracted from the medical insurance settlement platform across three counties in Hebei Province, covering January 2021 to July 2023. Ethical approval was obtained from the Institutional Review Board of the Institute of Medical Information, Chinese Academy of Medical Sciences, with anonymized data ensuring participant confidentiality.

### 2.2 Data sources

Individual inpatient data were obtained from the medical insurance settlement platform (health insurance claims) in three counties in Hebei province, covering all inpatient settlement data during the period from January 2021 to July 2023. According to the International Classification of Diseases, 10th revision (ICD-10) (15), IHD is classified under codes I20 to I25, which encompass conditions such as coronary heart disease, angina pectoris, and myocardial infarction. The analysis focused on the first hospitalization for IHD among residents with medical insurance who choose intra-provincial care rather than care in coordinated regions. The intra-provincial means the different cities in one province but not the coordinated region.

Demographic characteristics, including gender and age at admission, were included in the datasets. Hospital information comprised ICD-10 codes, primary diagnoses, hospital tiers, dates of visits and discharge, and lengths of stay. Expense-related data included total expenses, insurance reimbursement costs, and out-of-pocket expenditures. The individual-level data reflected various individuals at different points in time, representing a repeated cross-section. The policy intervention occurred at the group level.

### 2.3 Outcome variables

In the study, one hospitalizations visit means one single hospital admissions for the patient, not hospital encounters, e.g., ambulatory services provided in a hospital, brief hospital stays awaiting diagnostic information. The total expenses include the direct medical expenses incurred in the hospital, e.g., examination fee, drug cost, operation fee, bed fee, material fee, nursing fee, laboratory testing fee, etc.

### 2.4 Statistical analysis

The categorical variables were presented in terms of counts and percentages, and the chi-squared test was utilized to compare these variables. An interrupted time series analysis (ITSA) was employed to investigate the impacts of the policy on hospitalization visits and expenses for patients with IHD. ITSA is regarded as a quasi-experimental research design for establishing causality without randomization frequently applied to assess intervention effects (16), and often utilizes existing time-series data that have been collected routinely over an extended period. This method evaluates both the instantaneous and trend impacts of interventions by analyzing data collected at multiple time points before and after the implementation of the policy (17). This design accounted for autocorrelation and seasonal trends while isolating policy impacts from concurrent events, such as COVID-19 restriction adjustments. In the study, a weekly interval was used to analyze the change in trend (slope) and the change in level of the data indicators before and after the implementation of deregulation policy. The intervention point was designated as September 1st, 2021, marking the official implementation of deregulation policy. The ITSA regression model is specified as follows:


(1) $$\begin{array}{l} Y_{t}=\beta_{0}+\beta_{1}*\text{time}+\beta_{2}*{\text{intervention}}_{t}\\ +\beta_{3}*{\text{time after intervention}}_{t}+e_{t} \end{array}$$

In Equation 1, $Y_{t}\;$ represents the weekly outcome indicator for the period spanning from week 1of 2021 to week 26 of 2023, time is treated as a continuous variable that denotes the specific week; intervention is a binary indicator, taking the value of 1 following the implementation of the policy and 0 otherwise. Additionally, time after intervention is a continuous measure that counts the number of weeks post- intervention, assigning a value of 0 for periods preceding the intervention. Moreover, in this model, $\beta_{0}$ estimates the baseline level or intercept at time 0, $\beta_{1}$ estimates the trend or slope of change prior to the introduction of the intervention. $\beta_{2}$ estimates the instantaneous change following the intervention, and $\beta_{3}$ estimates the change in the trend or slope after the policy. Consequently, $\beta_{1}$ + $\beta_{3}$ reflects the actual trend of the outcome, representing the net effect of the policy intervention. $e_{t}$ is a random error term at moment t, which is not explained in the model. The Durbin–Watson (D-W) test was applied to assess first-order autocorrelation in the error terms, while the Paris-Winsten estimation method was employed to correct the D-W value. All data analyses were conducted using StataMP-64 V.17.0 software. The level of significance was set at 0.05.

## 3 Results

### 3.1 Characteristics of study population

This study included 8,522 IHD inpatients from three counties in Hebei province. Among these, 2,241 were admitted prior to the implementation of the policy, and 6,281 were admitted afterward. The sociodemographic characteristics of the IHD inpatients, categorized by gender, age, and tiers of hospitals before and after the policy implementation, are displayed in Table 1. Male patients accounted for 55.3%. Patients aged ≥60 years constituted the largest proportion of hospitalizations at both time points, accounting for 64.7% before the policy and 63.7% after its implementation. Overall, the age distribution was relatively consistent. The highest proportion of inpatients was treated at tertiary hospitals (tier 3). However, following the policy implementation, the percentage of admissions to tertiary hospitals decreased from 79.47 to 74.96%, while the percentage of patients admitted to primary and secondary hospitals (tier 1 and 2) increased. Additionally, the highest proportion of IHD inpatients had a length of stay of 7–9 days, which declined from 43.42 to 42.64% after the policy implementation. Conversely, the proportion of IHD inpatients with a length of stay of 7 days or fewer increased from 30.25 to 35.71% (*p* < 0.001).

**Table 1:** Basic characteristics of inpatients included before and after the deregulation policy.

Variables	Pre-policy (*n* = 2,241)	Post-policy (*n* = 6,281)	*χ*^2^	*p* values
Gender (*n*, %)			0.028	0.867
Male	1,243 (55.47)	3,471 (55.26)		
Female	998 (44.53)	2,810 (44.74)		
Age group (*n*, %)			0.961	0.811
<50 years	220 (9.82)	619 (9.86)		
50–59 years	571 (25.48)	1,661 (26.44)		
60–69 years	766 (34.18)	2,134 (33.98)		
≥70 years	684 (30.52)	1867 (29.72)		
Hospital tiers (*n*, %)			27.008	<0.001
Hospital tier 1	38 (1.70)	214 (3.41)		
Hospital tier 2	422 (18.83)	1,359 (21.64)		
Hospital tier 3	1781 (79.47)	4,708 (74.96)		
Length of stay (*n*, %)			30.359	<0.001
<7 days	678 (30.25)	2,243 (35.71)		
7–9 days	973 (43.42)	2,678 (42.64)		
≥10 days	590 (26.33)	1,360 (21.65)		
Total hospitalization expense per Visit (CNY) (*n*, %)			51.316	<0.001
<10,000	912 (40.70)	3,076 (48.97)		
10,000–20,000	616 (27.49)	1,608 (25.60)		
≥20,000	713 (31.82)	1,597 (25.43)		

Policy-health insurance deregulation policy.

**Figure 2:**
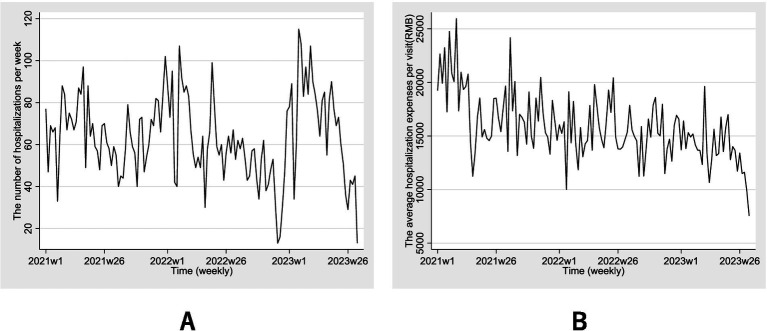
Weekly trends of hospitalization numbers **(A)** and average hospitalization expense **(B)** per visit from January 2021 to July 2023.

The proportion of IHD inpatients with total hospitalization expenses per visit of 10,000 CNY or less was the most substantial. After the policy implementation, this group increased from 40.70 to 48.97%, while the percentages of IHD inpatients with hospitalization expenses between 10,000 and 20,000 CNY and above both declined (*p* < 0.001). Figure 2 depicts the weekly trends of hospitalization numbers and average hospitalization expense per visit from January 2021 to July 2023. There are fluctuations that exhibit similar patterns for the weekly trends of hospitalization numbers, initially increasing and subsequently decreasing. There was a rapid increase beginning in 2023, followed by another downward trend. A consistent downward trajectory was observed in per-visit hospitalization expenses on a weekly basis.

### 3.2 The number of hospitalizations visits per week

**Figure 3:**
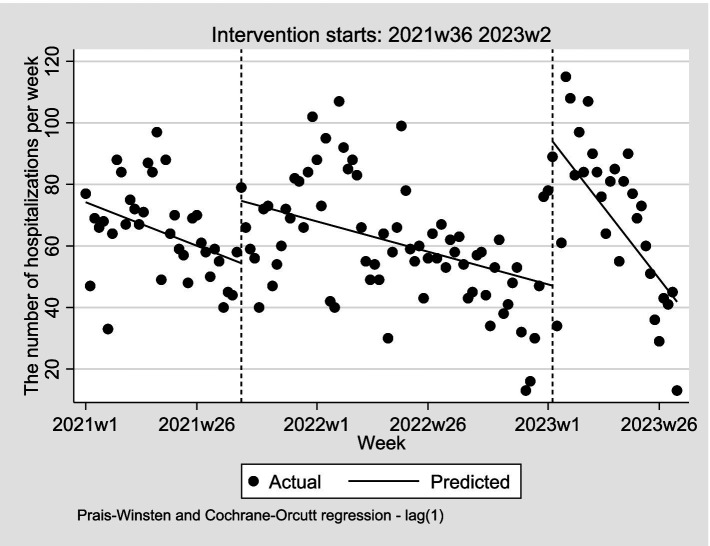
Weekly changes in number of hospitalizations, January 2021–July 2023.

Considering the transition from Category A to Category B management for COVID-19, effective from January 8, 2023, which may impact the accessibility of cross-regional medical services, a multiple treatment periods ITSA model was conducted, utilizing the weekly number of hospitalizations as the dependent variable. In this model, September 1, 2021 was considered as the initial time point for the implementation of insurance deregulation policy, and continued until the aforementioned date, signifying the lifting of COVID-19 interventions (18). The adjusted Durbin-Watson (DW) value was 1.84, indicating that the data satisfied the autocorrelation test requirements (19). Table 2 presents the model parameters. The results revealed that the estimated initial weekly number of hospitalizations was 74.21 (β_0_ = 74.21, *p* < 0.001), with a decreasing trend of 0.57 hospitalizations per week prior to the policy implementation (β_1_ = −0.57, *p* = 0.066). In the first week post-policy, a significant increase of 20.27 hospitalizations was observed (β_2_ = 20.27, *p* = 0.006), followed by a non-significant weekly increase of 0.17 hospitalizations (β_3_ = 0.17, *p* = 0.619). Additionally, immediately following the lifting of COVID-19 interventions, there was a significant increase of 46.92 hospitalizations in the first week (β_4_ = 46.92, *p* = 0.001), followed by a subsequent decrease of 1.47 hospitalizations in weekly trend (β_5_ = −1.47, *p* = 0.050). Figure 3 provides a visual presentation of these results.

**Table 2:** ITSA results for number of hospitalizations per week before and after the policy.

Variables	Coefficient	SE	t	*p* values	95% CI	DW
Number of hospitalizations per week						1.84
$\beta_{1}$ : baseline slope	−0.57	0.30	−1.86	0.066	(−1.17, 0.04)	
$\beta_{2}$ : level change after policy 1	20.27	7.20	2.82	0.006	(6.02, 34.52)	
$\beta_{3}$ : slope change after policy 1	0.17	0.35	0.50	0.619	(−0.51, 0.86)	
$\beta_{4}$ : level change after policy 2	46.92	13.80	3.40	0.001	(19.6, 74.23)	
$\beta_{5}$ : slope change after policy 2	−1.47	0.74	−1.98	0.050	(−2.94, 0)	
$\beta_{0}$ : baseline level	74.21	7.45	9.96	0.000	(59.47, 88.95)	

Policy 1- the health insurance deregulation policy.

Policy 2- the lifting of COVID-19 interventions from Category A to Category B management.

### 3.3 The hospitalization expenses and the insurance reimbursement cost

**Figure 4:**
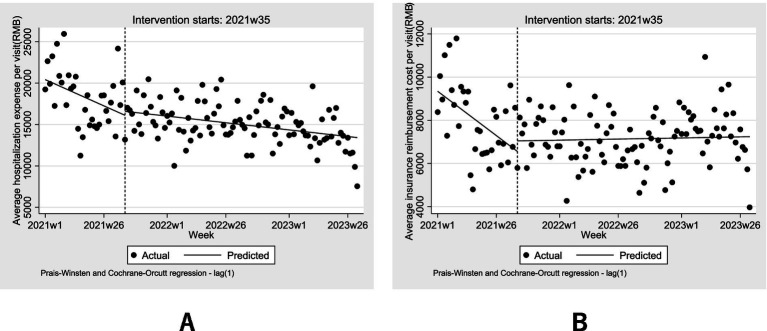
Weekly changes in the **(A)** average hospitalization expenses and **(B)** average insurance reimbursement cost 2021–2023.

Hospitalization expenses and insurance reimbursement costs are influenced by policy changes through health insurance reimbursement mechanisms, but the management level of COVID-19 does not have an impact. In this model, September 1, 2021 is designated as the initial time point for the implementation of the health insurance deregulation policy. The model parameters are summarized in Table 3. The adjusted D-W value ranged from 1.81 to 1.98, with values close to 2 indicating no autocorrelation (20). The results revealed that the estimated initial hospitalization expenses per visit was 20422.34 CNY (β_0_ = 20422.34, *p* < 0.001). Prior to the implementation of the policy, hospitalization expenses per visit exhibited a significant downward trend, decreasing by 126.71 CNY per visit weekly (β_1_ = −126.71, *p* = 0.037). In the first week following the implementation of this policy, hospitalization expenses per visit instantaneously increased by 478.43 CNY (β_2_ = 478.43, *p* = 0.723), followed by an increase of 94.67 (β_3_ = 94.67, *p* = 0.121) in weekly trend. After the implementation of the deregulation policy, total hospitalization expenses per visit maintained a downward trend of 32.04 per week (β_1_ + β_3_ = −32.04). Figure 4A provides a visual display of these results.

**Table 3:** ITSA results for the average hospitalization expense and insurance reimbursement cost.

Variables	Coefficient	SE	*t*	*p* values	95% CI	DW
Average hospitalization expenses per visit (CNY)						1.98
$\;\;\;\beta_{1}$ : baseline slope	−126.71	60.11	−2.11	0.037	(−245.63, −7.8)	
$\beta_{2}$ : level change after policy	478.43	1348.53	0.35	0.723	(−2189.47, 3146.34)	
$\beta_{3}$ : slope change after policy	94.67	60.73	1.56	0.121	(−25.48, 214.83)	
$\;\;\;\beta_{0}$ : baseline level	20422.34	1091.48	18.71	<0.001	(18262.98, 22581.71)	
Average insurance reimbursement cost per visit (CNY)						1.81
$\;\;\;\beta_{1}$ : baseline slope	−81.98	28.13	−2.91	0.004	(−137.63, −26.32)	
$\;\;\;\beta_{2}$ : level change after policy	490.74	603.07	0.81	0.417	(−702.37, 1683.84)	
$\beta_{3}$ : slope change after policy	83.93	28.55	2.94	0.004	(27.45, 140.42)	
$\beta_{0}$ : baseline level	9341.78	559.39	16.70	<0.001	(8235.09, 10448.48)	

The cost reimbursed by medical insurance include not only those covered by basic medical insurance, but also that covered by catastrophic medical insurance (also known as critical illness insurance or *Da Bing Yi Bao*), and the medical aid program (also refer to as medical financial assistance or *Yi Liao Jiu Zhu*). The insurance reimbursement cost per visit was 9341.78 CNY (β_0_ = 9341.78, *p* < 0.001), with a weekly decrease of 81.98 CNY prior to the implementation of the policy (β_1_ = −81.98, *p* = 0.004). In the first week following the implementation of the policy, insurance reimbursement costs per visit increased instantaneously by 490.74 CNY (β_2_ = 490.74, *p* = 0.417). After the health insurance deregulation policy, there was an increase of 83.93 (β_3_ = 83.93, *p* = 0.004) insurance reimbursement cost per visit in weekly trend. The trend shifted from a downward to an upward trajectory, increasing by 1.95 per week (β_1_ + β_3_ = 1.95). Although the instantaneous increase was statistically insignificant (*p* = 0.417), the upward trend is statistically significant (*p* = 0.004). Figure 4B provides a visual display of these results.

## 4 Discussion

This study provides the first interrupted time-series analysis evaluating both immediate and longitudinal impacts of health insurance deregulation policy on IHD hospitalization visits and expenses in Hebei Province. Our findings reveal three critical dynamics: (1) A significant immediate surge in hospitalization volumes post-policy (β = 20.27, *p* = 0.006) followed by stabilization, (2) Persistent downward trends in per-visit hospitalization expenses despite policy changes (pre-policy: −126.71 CNY/week, *p* = 0.037), and (3) A policy-driven reversal in insurance reimbursement costs from decreasing (−81.98 CNY/week) to increasing trajectories (+1.95 CNY/week, *p* = 0.004). These patterns carry important implications for chronic disease management in transitioning healthcare systems.

The immediate surge in hospitalizations is consistent with behavioral economics theories predicting increased care-seeking when administrative barriers are reduced (20). However, the attenuation of this effect over time (β_3_ = 0.17, *p* = 0.619) suggests that pent-up demand from pre-policy system friction constituted the primary driver, rather than sustained increases in disease incidence. In this regard, the estimation results align with those of Xu (21), Mckee and Belcher (22). At the national level, the total number of URRBMI patients seeking healthcare in cross regions in 2022 (37.51 million visits) was lower than the number in 2021 (43.18 million visits) (23, 24). However, in 2023, the number of URRBMI patients seeking cross-regional healthcare rose to 82.14 million visits, surpassing the combined total from 2021 and 2022 (15). Consequently, the change hospitalizations visits for cross-regional healthcare from the three counties is generally consistent with the national trend.

Regarding hospitalization expenses, the findings of the present study diverged from the initial hypotheses. While hospitalization visits rose significantly, per-visit expenses continued their pre-existing decline (post-policy: −32.04 CNY/week), likely reflecting concurrent payment reforms promoting cost containment. The overall downward trend in hospitalization expenses per visit may be attributed to reforms in medical insurance payment methods, the severity of IHD, and the phenomenon of hospitalization decomposition resulting from these payment reforms (25). In terms of total medical expenses, the analysis results is different from that of Xu (21), which encompassed all the inpatient claim data. As for more serious medical conditions such as IHD, the finding is consistent with that of Aviva (26), which indicated the effect of cost sharing on the level of inpatient spending is consistently small and generally insignificant, due to the less price sensitivity.

Additionally, the rising reimbursement costs (1.95 CNY/week, *p* = 0.004) despite stable hospitalization expenses suggests systemic shifts in insurance claim patterns rather than clinical cost inflation - potentially indicating improved billing compliance or altered service mix documentation post-policy. Compared with the studies of Andricus and Tang (27) and Xu (21), which suggested that cross-border patient mobility reduced the reimbursement costs or had an insignificant impact, the implementation of the deregulation policy has eliminated discrepancies in reimbursement rates, resulting in an increase compared to previous policies.

Our results mitigate concerns regarding unsustainable financial pressures on insurance funds from cross-regional care. The moderate reimbursement cost increases (1.95/week) contrast sharply with projections from European models (4), likely reflecting China’s tiered reimbursement structure and persistent intra-provincial price controls. This aligns with Yangtze Delta outpatient studies showing stable utilization post-deregulation (7), suggesting that regional economic integration may mitigate financial risks through economies of scale.

While employing robust ITSA methodology to isolate policy effects, several limitations warrant consideration. First, the single-province focus may limit generalizability, though Hebei’s role as a Beijing-Tianjin-Hebei integration hub enhances relevance. Second, aggregated expense data preclude analysis of clinical drivers (e.g., medication vs. procedure costs). Third, unmeasured confounders like parallel payment reforms may influence cost trajectories. Future multi-province studies incorporating detailed cost structures and patient-reported outcomes would strengthen evidence.

## 5 Conclusion

This study demonstrates that the removal of record-filing requirements (health insurance deregulation policy) significantly enhanced healthcare accessibility for IHD patients in Hebei Province, as evidenced by an immediate 20.27 surge in weekly hospitalizations (*p* = 0.006) following the policy implementation. Additionally, the policy reversed the previously declining trend in insurance reimbursement costs, resulting in a sustained upward trend trajectory (1.95 CNY/week, *p* = 0.004). Despite concerns about escalating costs, per-visit hospitalization expenses maintained their pre-policy declining trajectory (−32.04 CNY/week), highlighting the policy’s success in decoupling utilization growth from increased financial burden. These findings highlight the deregulation policy’s dual role in addressing systemic inequities—by reducing administrative barriers to cross-regional care and aligning with broader payment reforms aimed to cost containment. To optimize these gains, we emphasize the urgency of decentralizing tertiary hospital resources through medical consortiums and telemedicine networks, thereby retaining patients within coordinated regions without compromising the quality of care. These insights provide an evidence-based framework for refining insurance mechanisms for aging populations, particularly in tailoring reimbursement policies according to disease severity and enhancing primary care capacities for chronic disease management.

## Data Availability

The data analyzed in this study is subject to the following licenses/restrictions: the data used cannot be shared unless applicants secure the relevant permissions. Requests to access these datasets should be directed to cui.yueying@imicams.ac.cn.

## Footnotes

^1^The Yangtze River Delta region is located in the eastern part of China. It includes parts of Shanghai, Jiangsu, Zhejiang, and Anhui provinces. In this context, the insurance deregulation policy is among different provinces.
